# REMEMProt: a resource of membrane-enriched proteome profiles, their disease associations, and biomarker status

**DOI:** 10.26508/lsa.202302443

**Published:** 2024-05-07

**Authors:** Anjana Aravind, Revathy Nandakumar, Mukhtar Ahmed, Mahammad Nisar, Akhina Palollathil, Anagha Kanichery, Sourav Sreelan, KP Munavvar Sinan, Rex Devasahayam Arokia Balaya, Manavalan Vijayakumar, Thottethodi Subrahmanya Keshava Prasad, Rajesh Raju

**Affiliations:** 1 Center for Systems Biology and Molecular Medicine, Yenepoya Research Centre, Yenepoya (Deemed to be University), Mangalore, India; 2 Department of Zoology, College of Science, King Saud University , Riyadh, Kingdom of Saudi Arabia; 3 Centre for Integrative Omics Data Science (CIODS), Yenepoya (Deemed to be University), Mangalore, India; 4 Yenepoya Institute of Technology, Yenepoya (Deemed to be University), Mangalore, India; 5 Department of Surgical Oncology, Yenepoya Medical College, Yenepoya (Deemed to be University), Mangalore, India

## Abstract

The differential expression of plasma membrane proteins is integrally analyzed for their diagnosis, prognosis, and therapeutic applications in diverse clinical manifestations. Necessarily, distinct membrane protein enrichment methods and mass spectrometry platforms are employed for their global and relative quantitation. First of its kind to explore, we compiled membrane-associated proteomes in human and mouse systems into a database named, Resource of Experimental Membrane-Enriched Mass spectrometry–derived Proteome (REMEMProt). It currently hosts 14,626 proteins (9,507 proteins in *Homo sapiens*; 5,119 proteins in *Mus musculus*) with information on their membrane-protein enrichment methods, experimental/physiological context of detection in cells or tissues, transmembrane domain analysis, and their current attribution as biomarkers. Based on these annotations and the transmembrane domain analysis in proteins or their binary/complex protein–protein interactors, REMEMProt facilitates the assessment of the plasma membrane localization potential of proteins through batch query. A cross-study enrichment analysis platform is enabled in REMEMProt for comparative analysis of proteomes using novel/modified membrane enrichment methods and evaluation of methods for targeted enrichment of membrane proteins. REMEMProt data are made freely accessible to explore and download at https://rememprot.ciods.in/.

## Abstract (precis)

A resource of experimental specific membrane-enriched mass spectrometry–derived proteome.

## Graphical Abstract

**Figure:**
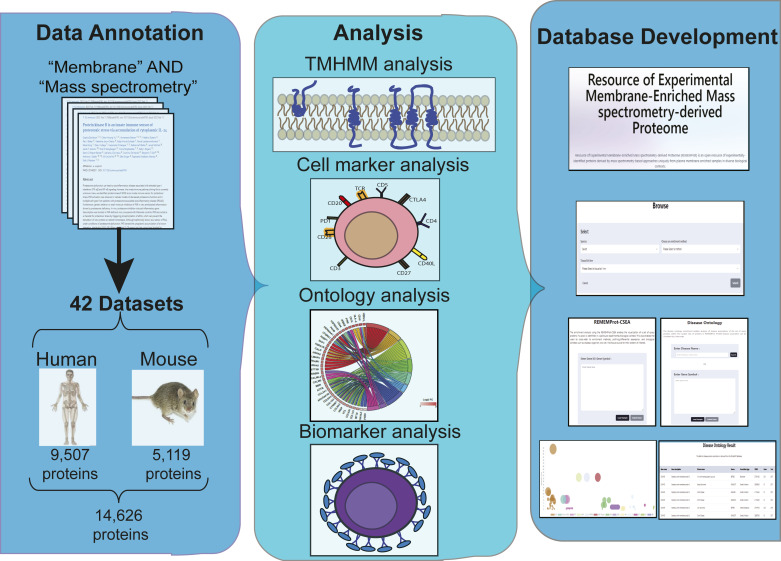

## Introduction

Membrane proteins are an essential component of cellular functions, such as signal transduction, ion transport, cell motility, and cell adhesion, among others (Almen et al, 2009). In addition, in view of their biomedical applications, they are the major targets for drug discovery, together constituting 50% of the current drug targets (Fagerberg et al, 2010). Along with the secreted proteins, membrane proteins serve as cellular and tissue markers for theranostic approaches. The membrane proteins are closely associated with lipids in the plasma membrane and harbor both hydrophilic and hydrophobic regions. This amphipathic nature of membrane proteins contributes to their heterogeneous and unique functions (Mirza et al, 2007). Consequently, extraction, solubilization, and characterization of these proteins have become a major challenge in the field of membrane proteomics (Vuckovic et al, 2013). To overcome these limitations, various strategies such as enrichment, solubilization, separation, and digestion steps are employed for effective detection or quantification of the membrane proteins (Alfonso-Garrido et al, 2015). However, despite the availability of multiple enrichment and extraction methods, the complete extraction of membrane proteins could not be ensured as each method differs in its enrichment efficiency and purity of the resulting membrane fraction (Lehner et al, 2003).

The advancements in mass spectrometry–based techniques have enabled comprehensive protein identification and quantification on a global scale, leading to the discovery and validation of actionable biomarkers or therapeutic targets (Sadowski et al, 2008; Subbannayya et al, 2015; Radhakrishnan et al, 2016). The high-throughput analysis and simultaneous detection of multiple proteins using mass spectrometry have facilitated membrane proteome assignment and quantification with high sensitivity and selectivity, even at low abundance (Schey et al, 2013). Although the detection and quantification of membrane proteins are crucial for biomedical applications, there are many challenges in choosing the suitable enrichment method to isolate and identify membrane proteins. Currently, the analyzed membrane proteome data scattered across multiple datasets in the literature makes it difficult for the researchers to refer to multiple enrichment strategies that significantly enrich specific proteins of interest. This necessitates a reference resource of membrane proteome for the biomedical community that can offer information regarding the proteins identified from distinct cellular sources employing various membrane protein enrichment methods along with their biological context of identification.

Several online database systems are now available to organize information on proteins (Kim et al, 2014; Nanjappa et al, 2014; Raju et al, 2014; Ridha et al, 2023), and explicitly for plasma membrane proteome, the data emphasize their structures and models and prediction algorithms for determining protein topology (Krogh et al, 2001; Bernsel et al, 2009; Liu et al, 2020). In this regard, an assembled database for the experimentally derived membrane-enriched proteome would help in the evaluation of the properties of integral and peripheral proteins using these approaches. Toward this, we manually curated and compiled the proteins identified from various cell types or tissues across studies using mass spectrometry–based proteomic approaches upon employment of distinct membrane protein enrichment methods into a resource named Resource of Experimental Membrane-Enriched Mass spectrometry–derived Proteome (REMEMProt). We believe that REMEMProt will serve as a primary reference platform for the mass spectrometry–derived membrane proteome. It would also help the researchers evaluate enrichment methods for a set of membrane proteins of their choice identifiable using mass spectrometry in mammalian systems.

## Results and Discussion

The membrane protein databases serve as a comprehensive repository for the structural, sequence, biological, and functional annotation of the membrane proteins. These databases provide a platform for search, analysis, and visualization of the information on membrane proteins facilitating research toward linking the molecular and functional annotations to the pathophysiology. To date, several membrane protein databases are available, characterizing the structural features, transmembrane topology, and cellular localization. A notable example of such a database is Membranome, a repository offering structural information on single-pass TM proteins. It also incorporates a computational method called TMDOCK to model the homodimers of TM α-helices (Lomize et al, 2017). Another example is TOPDB: topology data bank of transmembrane proteins, a TM database containing experimentally derived topology information and its structural information (Tusnady et al, 2008). PerMemDB is another database exclusively for peripheral membrane proteins from eukaryotes (Nastou et al, 2020). However, a database featuring the experimental analysis on the detection of membrane proteins and its explored utility focusing on their application in translational research is limited. In this underlying context, we developed a robust catalog of membrane proteins incorporating their functional attributes including topology, ontology, and marker utility status. Contrary to the currently available resources, the REMEMProt serves as a stand-alone reference platform for the membrane proteins detected and differentially expressed undertaking distinct membrane enrichment methods. This repertoire also serves as a resource for evaluation and reference for the plasma membrane enrichment techniques suitable for the protein set of the user’s choice that can be identified using mass spectrometry–based approaches.

### Database content and functionalities

#### REMEMProt

The REMEMProt database is an integrative resource of proteins extracted through diverse membrane enrichment approaches coupled to their identification using mass spectrometry–based proteomics. The database currently hosts information on 14,626 membrane proteins distinguished based on cell line and tissue sources in *Homo sapiens and Mus musculus* representing different experimental and disease conditions, of which 4,096 and 1,457 proteins in human and mouse, respectively, have been reported in more than two studies. Across 40 studies, a total of 9,507 unique proteins were compiled from *H. sapiens* and 5,119 proteins from *M. musculus*. These studies were envisioned for the comparative analysis of disease biomarkers or therapeutic targets, distinct membrane protein enrichment methods, or evaluation of novel strategies for enhanced detection/identification of membrane proteins. The REMEMProt is made available free online at (https://rememprot.ciods.in/) with a user-friendly interface for search, analysis, and batch query.

**Figure 1.**
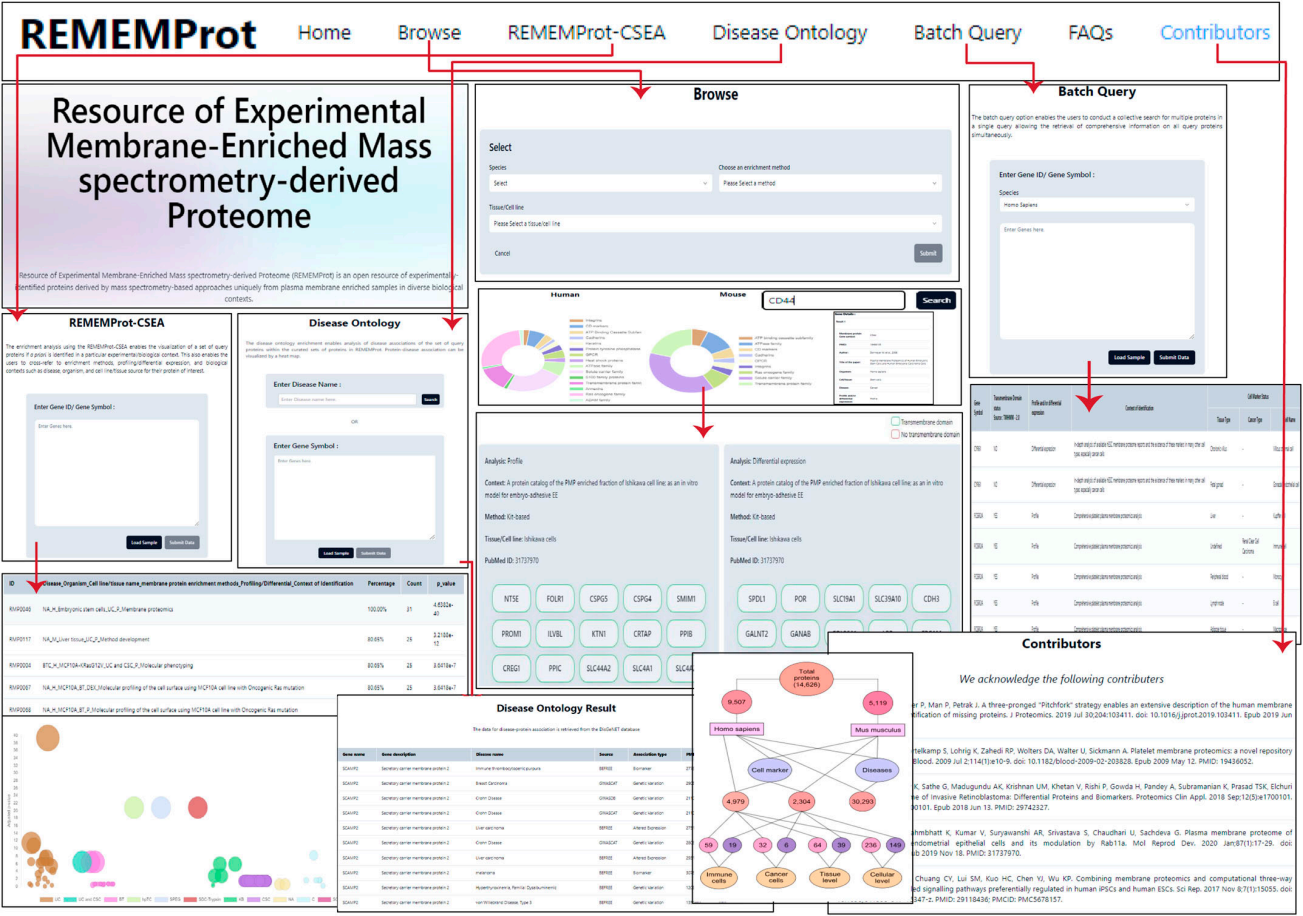
A detailed navigation through the REMEMProt. The REMEMProt database has features that enable the search of proteins by their gene symbol based on the organism, cellular source, and extraction method. The enrichment tool is facilitated to visualize the biological and functional contexts of the user proteins of interest, and the disease ontology tool is for the visualization of disease-to-gene association. A batch query option to retrieve the information for a set of query proteins.

The “Browse” page enables serial query selection by species, membrane protein enrichment method, and cell line/tissue type to visualize the list of proteins under the specific category along with their transmembrane, biomarker, and cell marker status. In addition, we have incorporated an option for a single protein query that can be searched using either gene symbol or gene ID. This enables the retrieval of information about the study details from where the protein was curated and annotated and its transmembrane status. For enhanced query and cross-database reference, the REMEMProt is integrated with analysis tools for the enrichment of a list of user-provided proteins by a batch query against REMEMProt (REMEMProt-CSEA) and also visualize the REMEMProt proteins disease associations (Disease Ontology analysis) (Fig 1). Furthermore, the batch query provides access to the users to retrieve comprehensive information on the multiple query proteins with their transmembrane status, subcellular localization, binary and complex interactors among transmembrane proteins, and cell marker status. Also, we have enabled an additional query search option within the batch query that redirects toward the cancer surfaceome atlas (Hu et al, 2021). This search provides access to the users to the expression levels, structural characteristics, and features of surface proteins in different cancer types.

### Transmembrane domain and functional analysis of REMEMProt proteins

**Figure 2.**
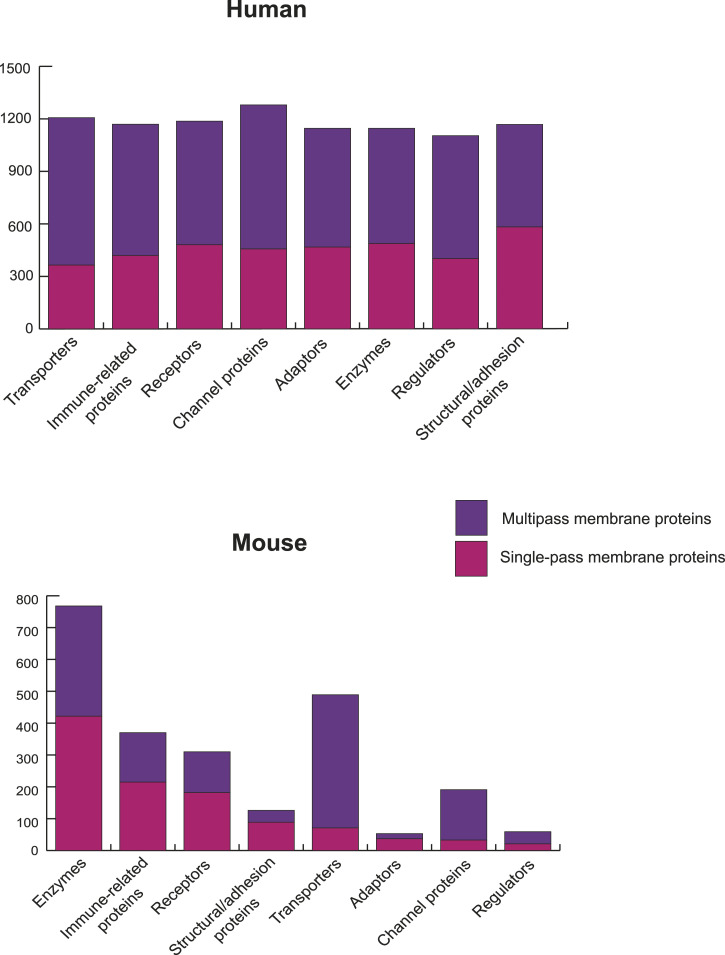
The Gene Ontology–based classification of single-pass and multi-pass transmembrane proteins. The graph illustrates the classification of different classes of proteins from Gene Ontology analysis into single-pass and multi-pass based on the transmembrane domains among proteins of human and mouse.

Proteins assembled in REMEMProt were analyzed for predicted transmembrane domains using TransMembrane prediction using Hidden Markov Models (TMHMM) software (Krogh et al, 2001). A large number of proteins in REMEMProt contain transmembrane domains in one or more of the protein isoforms and hence have a potential association with the plasma membrane. Proteins as many as 3,103 out of 9,507 and 2,618 out of 5,119 in human and mouse systems, respectively, characteristically harbored one or more transmembrane domains. This included the proteins with 1,282 single-pass and 1,653 multi-pass transmembrane proteins in humans and 1,327 single-pass and 1,446 multi-pass transmembrane proteins in mouse. Toward the classification of proteins based on their molecular functions, Gene Ontology (GO) analysis led to the assignment of proteins into their respective functional classes including receptors, immune-related proteins, transporters, channel proteins, enzymes, adaptors, regulators, and structural/adhesion-related proteins (Table S1). The occurrence of predicted single-pass and multipass transmembrane topology of proteins in these molecular function categories visualized a variation across the species (Fig 2). In addition to the TMHMM analysis for the transmembrane status of the proteins, we also retrieved the transmembrane proteins within the binary and complex interactors of the target membrane proteins. This aids in predicting potential protein interactions within the transmembrane proteins.

Table S1 Table representing the classification of the curated proteins in the REMEMProt based on the molecular functions for human and mouse systems.

**Figure 3.**
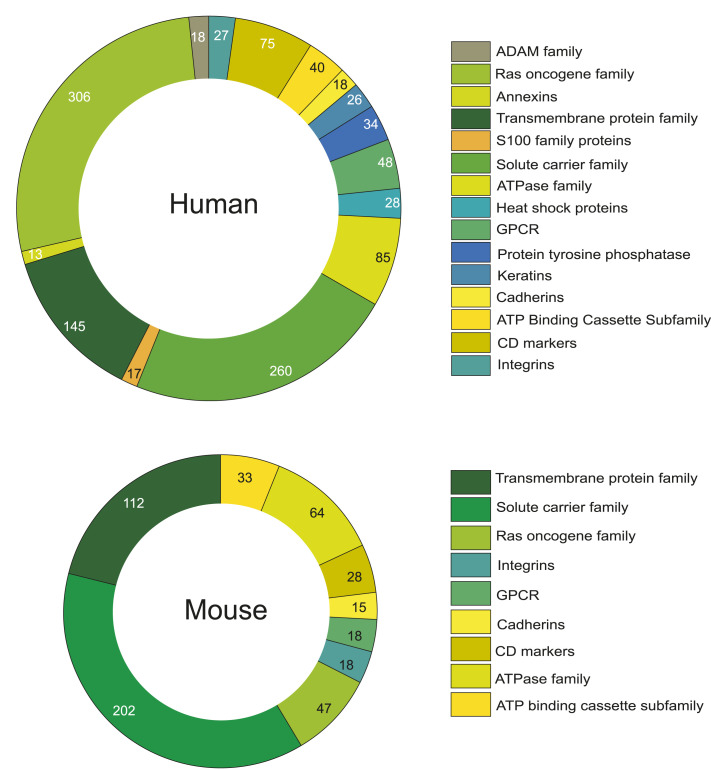
The distribution of protein to different protein families. The graph represents the distribution of the proteins from human belonging to different classes of protein families in human and mouse systems.

Moreover, it was observed that a significant proportion of proteins assembled in the database belonged to multiple protein families. The Ras oncogene family is one such class of GTPases that are plasma membrane–localized proteins (Hancock & Parton, 2005). The solute carrier family and transmembrane protein family are other groups consisting of membrane transport proteins and integral membrane proteins with at least one transmembrane segment, respectively (Marx et al, 2020; Pizzagalli et al, 2021). Cluster of differentiation (CD) proteins, the cell surface markers of immunophenotyping, is another class of proteins (Kalina et al, 2019). The other classes include G-protein coupled receptors, ATPases, annexins, cadherins, integrins, the S100 protein family, and more (Fig 3).

### REMEMProt cross-study enrichment analysis (REMEMProt-CSEA)

REMEMProt cross-study enrichment analysis provides a comprehensive view of all the proteins collected from various literatures and assembles their characteristic study-centric information. This leads to the adaptive visualization of proteins, given a priori identification within a specific experimental or biological context. We structured these attributes into tailor-made annotation terms, as outlined in Table 1, facilitating users to cross-refer all attributes and make comparisons even when sourced from the diverse literature. The results are highlighted in an interactive bubble plot generated according to the close associations of query proteins to that of annotated enriched terms. The plot illustrates the enrichment results of the user query proteins according to the method of membrane protein extraction and adjusted *P*-value. The x-axis shows the types of extraction methods and the corresponding enrichment *P*-value in the negative log_10_ scale is illustrated on the y-axis. Each bubble on the plot represents the count of proteins that comes under a unique functional term.

**Table 1.** Table representing the examples of REMEMProt CSEA enrichment terms and their corresponding IDs.

ID	Enrichment term
RMP0072	NA_H_Monocytes_BT_P_Surface proteome
RMP0004	BTC_H_MCF10A-KRasG12V_UC and CSC_P_Molecular phenotyping
RMP0138	RB_H_Tissue_KB_DEX_Biomarkers
RMP0030	H_ESC_NA_P_Available ESC membrane proteome reports

NA, not available; H, human; BT, biotinylation; P, profiling; BTC, breast cancer; UC, ultracentrifugation; CSC, cell surface capturing; RB, retinoblastoma; KB, kit-based method; DEX, differential expression; ESC, embryonic stem cells.

Alongside the bubble plot, a detailed table is provided with extensive information on each gene with its associated enrichment. This helps the user to easily understand which method of extraction is mostly adopted for membrane protein extraction of query protein and their biological contexts. This largely reduces the time required for the user to understand the background of their proteins, and the same will be provided in just one click. Also, a concatenated approach (“*Disease_Organism_Cell line/tissue name_Membrane protein enrichment method_Profiling/Differential expression_Context of Identification*”) that collectively represents all the biological contexts in one go is an ideal way to represent the complex information collected from various literatures. All the query proteins that are enriched under different annotation terms were given unique IDs and represented as each bubble in the plot. The tool allows the user to identify overrepresented proteins associated with the query list compared with the background protein set enriched for their particular biological contexts. The count is calculated based on the number of query hits belonging to the particular enrichment term and percentage coverage intending the enrichment percent of query hits out of total query proteins.

### Disease ontology and biomarker status of REMEMProt proteins

The disease ontology analysis module allows the users to perform the disease ontology enrichment analysis of the proteins searched against the REMEMProt data. This enrichment analysis can be performed for the enlisted proteins in the database from human and mouse, systems as supported by the DisGeNET database (Pinero et al, 2017). This analysis enriches the known association of specific membrane proteins to various disease conditions. The data are substantiated by a score ranging from 0 to 1 that considers supporting literature evidence, model organisms, and level of curation. It also helps to understand the inter-link between proteins based on their association with multiple diseases and their co-expression profiles in disease conditions as diagnostic markers and therapeutic targets. The current data-dependent analysis of the human protein–disease association indicated that most of the membrane proteins are associated with multiple types of cancers. To investigate the biomarker status of the REMEMProt protein data, a search against the biomarker database Biomarker Knowledgebase for Animals was carried out (Wang et al, 2024). The data revealed the potential role of the membrane proteins in various disease conditions such as diagnostic, prognostic, predictive, and therapeutic factors.

### Analysis of membrane proteins based on enrichment methods

**Figure 4.**
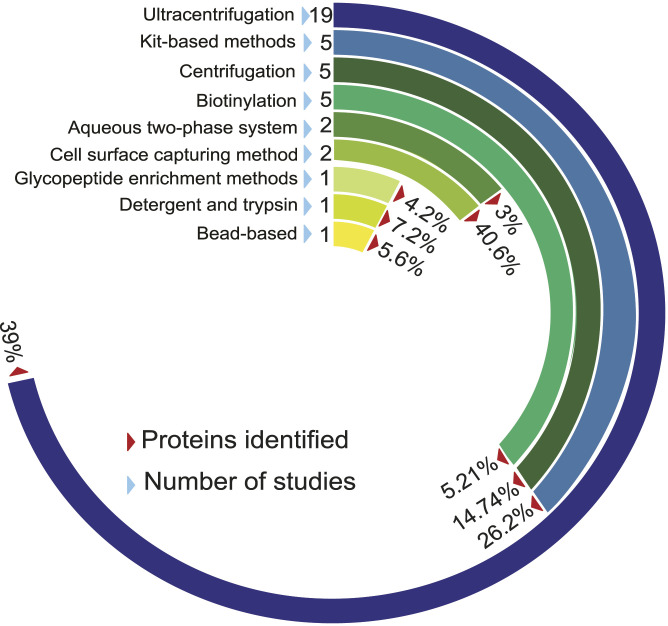
The frequency of different membrane protein enrichment methods and the number of proteins enriched from each method. The circular bar plot depicts the occurrence of different membrane protein enrichment methods used within the datasets in REMEMProt. In addition, it displays the percentage of the number of studies that used the enrichment methods and the proteins identified. Abbreviations: CSC, cell surface capturing.

Several analytical and enrichment methodologies have been used for effective membrane protein extraction and subsequently, their detection. These extraction methods are based on exploiting their physicochemical properties including the differential density of membrane proteins, high hydrophobicity, and negative charge across the membrane (Pauwels et al, 2022). Across the curated datasets in REMEMProt, multiple plasma membrane protein extraction methods have been followed. Among the following methods such as ultracentrifugation, cell surface capturing method, kit-based, biotinylation, glycopeptide enrichment, and aqueous two-phase partitioning, most of the studies have undertaken the ultracentrifugation method (Fig 4). Each method for membrane-protein extraction/enrichment varies based on its unique principle. Consequently, the cell surface capturing (CSC) technology selectively enriches surface proteins that are N-glycosylated (Glyco-CSC, Cys-Glyco-CSC), or have an extracellularly exposed and conformationally available lysine (Lys-CSC) (Bausch-Fluck et al, 2012). Similarly, biotinylation targets the plasma membrane proteins composing extracellular domains of integral and membrane-associated proteins (Li et al, 2021).

**Figure 5.**
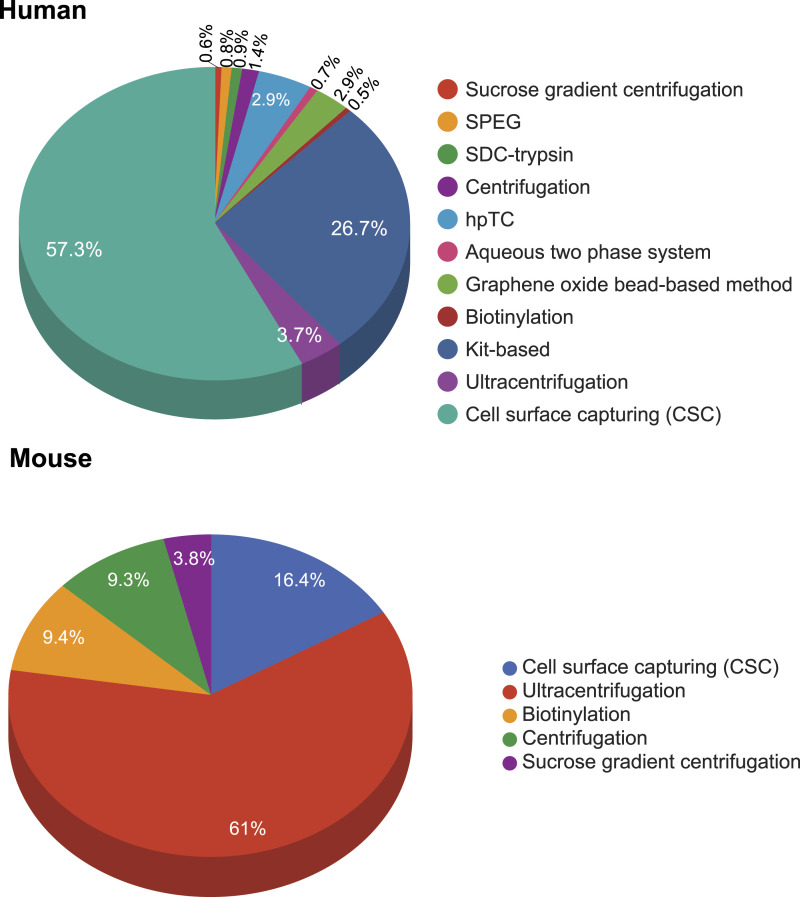
The count of unique proteins identified through various membrane protein enrichment methods. The pie diagram represents the number of unique proteins detected from distinct membrane protein enrichment methods in human and mouse.

**Figure 6.**
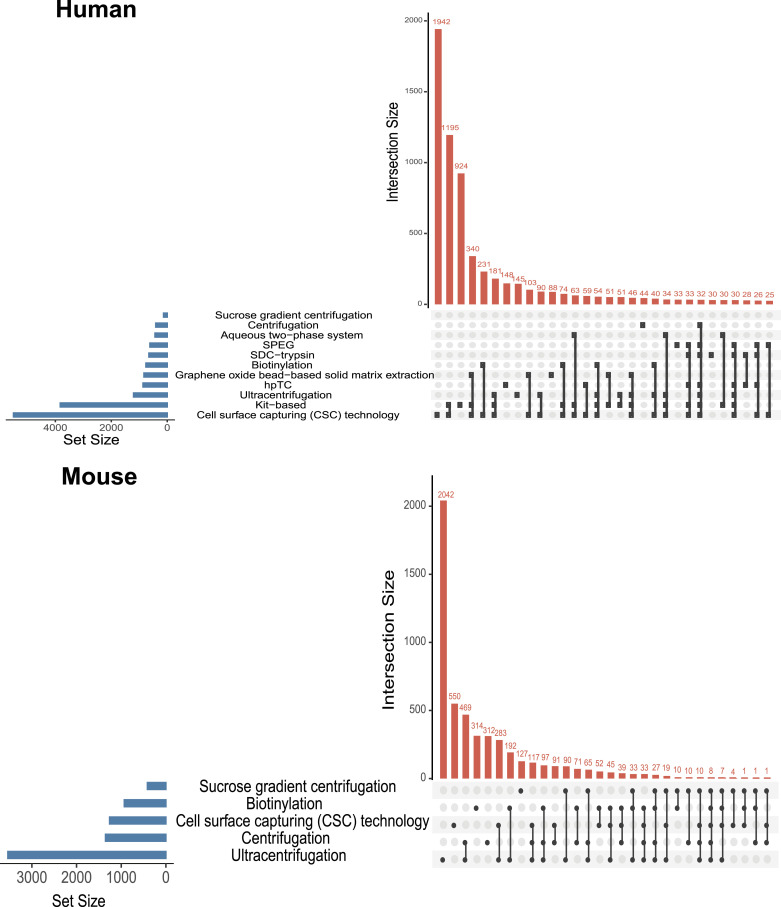
The number of proteins that overlap between various membrane protein enrichment methods. The upset plot illustrating the number of common proteins among different membrane protein enrichment methods in human and mouse.

For the REMEMProt datasets, we categorized the proteins that were identified based on the distinct enrichment methods. Although limited to the current datasets and their identification using mass spectrometry platforms, we identified certain sets of proteins that were uniquely enriched using a particular membrane protein isolation method (Fig 5). Curiously, we also enlisted the proteins that overlapped across multiple enrichment methods used in diverse experimental contexts from human, and mouse (Fig 6) (Table S2).

Table S2 Table representing the list of unique and common proteins detected from distinct membrane protein enrichment methods from human and mouse systems.

### Distinct cell types and cellular markers

The study focuses on developing a comprehensive resource for proteins potentially associated with plasma membranes based on its experimental context of identification. The curated datasets belonged to the studies on multiple disease conditions using different biological samples of both cellular and tissue sources, including PBMC comprising various immune cells from cancer patients, tumor cell line models, platelets, embryonic stem cells, tumor spheres, epithelial tissues, brain tissues and, xenograft models. This study enlists the proteins enriched using membrane protein extraction methods from these cellular sources. Furthermore, the annotated data for human and mouse within the database was compared with the data provided in the CellMarker 2.0 database to detect cellular markers for various cell and tissue types (Zhang et al, 2019). This enhances the users to query the specific cell markers within the REMEMProt database as a key to choose the enrichment methods to analyse the cell type of their interest. The result enlists 4,979 membrane proteins as cell markers for 226 cell types including 59 immune cells, 64 tissue types, and 32 cancer conditions in human, and 2,304 membrane proteins were found as markers for 145 cells including 19 immune cells, 39 tissue types, and 6 cancer conditions in the mouse (Table S3).

Table S3 The table shows the enrichment of proteins for the cellular markers using the CellMarker DB data from human and mouse.

The REMEMProt database is a valuable resource for the biomedical research community focusing on membrane proteins. Our database provides information on membrane proteins identified through multiple extraction methods and proteomic approaches from various cell lines and tissues, including their transmembrane, biomarker, and cell marker status. REMEMProt also incorporated with an efficient custom-based analysis tool for enrichment and disease ontology analyses to enable users to investigate gene–disease associations and protein expression in different biological contexts. It also helps to understand the inter-operability between proteins based on their association with multiple diseases and their co-expression profiles in disease conditions as diagnostic markers and therapeutic targets. REMEMProt will facilitate basic research by proving its usefulness in understanding the biology of membrane proteins and enabling the selection of suitable enrichment methods. In the future, we are committed to constant updation and also the annotation of membrane protein sequence and structural level information into REMEMProt. We solicit the support and active participation of the scientific community toward the efficient annotation of membrane proteins into REMEMProt. We believe that REMEMProt will also help basic researchers investigating the properties and the biological role of membrane proteins and enable the selection of suitable enrichment methods for their proteins of interest.

## Materials and Methods

### Data collection and annotation strategy

**Figure 7.**
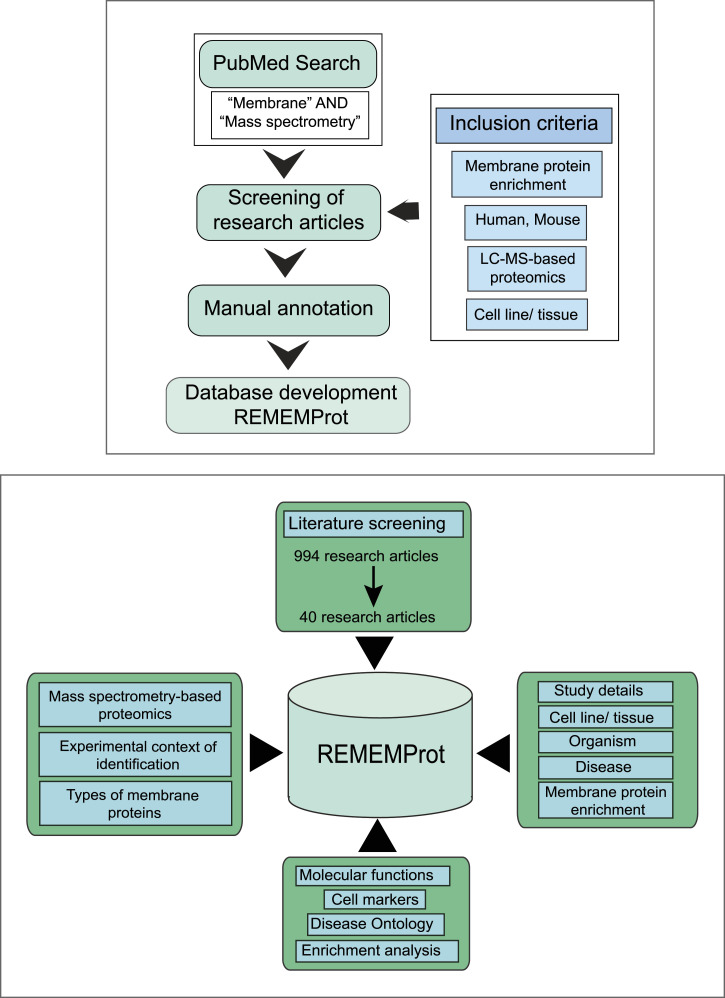
Workflow for the development of the REMEMProt. The mass spectrometry–derived proteomic data was retrieved from each dataset related to membrane proteomics based on the inclusion criteria. The retrieved data were then subjected to TMHMM analysis, cell marker analysis, and ontology analysis. Furthermore, these data were then used for the development of the REMEMProt database.

The REMEMProt database is aimed to encompass the information on experimentally derived membrane protein as a primary resource. Hence, we conducted systematic literature queries and annotation criteria to maximize the availability of the data. Toward this, we screened research articles using the search terms “plasma membrane proteins” AND “mass spectrometry.” Currently, the proteome annotations are restricted to mammalian systems (human and mouse). We compiled both profiling and differential proteome datasets, including information on the cell lines/tissues and membrane protein enrichment methods employed in the studies (Thomas et al, 2014). In addition, we also documented the experimental context of the identification of the membrane proteins from each study. The classification of transmembrane proteins within the data was based on a membrane protein topology prediction software TMHMM (Krogh et al, 2001). The workflow for the screening, assembly, and development of REMEMProt is provided in Fig 7.

### Database structure

REMEMProt, the online database was developed using the Django web development framework in Python. The database is composed of the back-end and the front-end components. The back-end of REMEMProt is a Django application served by the NGINX (Engine-X) server, and it employs a My Structured Query Language database management system for storing and managing data. This combination of technologies provides robust and scalable solutions for data management. The front end of REMEMProt is designed primarily with HyperText Markup Language, Cascading Style Sheets, and JavaScript, and it has been optimized for user-friendliness and responsiveness. To ensure a seamless user experience, the REMEMProt front-end has been developed with an intuitive and accessible design that facilitates easy access to information.

### Analysis of the membrane proteins in REMEMProt

#### Functional enrichment analysis

To compile the list of transmembrane proteins we ensure all protein isoforms of genes are included for transmembrane domain analysis. We converted the gene IDs to RefSeq accessions using the bioDBnet online tool (Mudunuri et al, 2009) and then used the Entrezpy python library to fetch FASTA sequences for the RefSeq accessions. To identify transmembrane proteins, we searched for proteins with any of their isoforms containing a transmembrane domain. We used the TMHMM software version 2.0 to detect transmembrane helices within the FASTA sequences (Krogh et al, 2001).

Furthermore, these proteins were enriched for their molecular function(s) using g: Profiler (Reimand et al, 2016). The Disease Ontology enrichment analysis was carried out to annotate the enlisted membrane proteins in the context of diseases. This enrichment analysis characterizing the protein–disease association was structured utilizing the data from the DisGeNET database for disease ontology (Pinero et al, 2017). The enlisted proteins from human, and mouse, can be distinctively subjected to disease ontology analysis to visualize their associated diseases. Similarly, the biomarker status of the proteins was fetched from the Biomarker Knowledgebase for Animals database (Wang et al, 2024) Furthermore, the proteins were also enriched for the information on cellular markers using the CellMarker 2.0 database (Zhang et al, 2019).

### REMEMProt-CSEA

REMEMProt-CSEA provides a collective idea about how the query proteins are enriched according to their method of extraction based on all the literature collected for this study. All the proteins in the database were mapped to their biological contexts by expert manual curation. The enlisted proteins and their association with various biological contexts such as disease, cellular source, and membrane protein extraction techniques were comprehensively grouped and mapped to each protein. The input protein list can be composed of an HGNC gene symbol. The REMEMProt-CSEA is implemented based on the statistical significance using a standard method of Fisher’s exact test (Sprent, 2011). Fisher’s exact test was employed to quantitatively assess the significant association between a set of user input genes and its unique enrichment terms. A 2 × 2 contingency table was built with the user input data and all the datasets in the database as a matrix given below:

[x n − x]

[N − x M − (n+N)+x]

x = the number of hits from the user’s query list.
N = the total number of proteins in the user’s query list.
n = the total number of proteins that belong to a particular method of membrane protein extraction.
M = the total number of proteins in the database (species-specific).

The *P*-value represents the significance of the proteins in the query list that are enriched based on their common method of membrane protein extraction, which was calculated using the Python package *scipy.stats.fisher_exact*. For the best possible choices, the parameter is set to “two-sided,” which implies the odds ratio of the underlying population is not one. An interactive bubble plot was used to visualize the CSEA results implemented using a JavaScript library, Chats.js. The bubble plot represents the data based on the membrane protein extraction methods indicated with different colors.

## Supplementary Material

Reviewer comments
